# FOXR2 in cancer development: emerging player and therapeutic opportunities

**DOI:** 10.32604/or.2024.052939

**Published:** 2025-01-16

**Authors:** PIAO YANG, MOHSEN SHEYKHHASAN, REZA HEIDARI, MOHSEN CHAMANARA, PAOLA DAMA, AMIRHOSSEIN AHMADIEH-YAZDI, HAMED MANOOCHEHRI, HAMID TANZADEHPANAH, HANIE MAHAKI, NASER KALHOR, ASHKAN DIRBAZIYAN, SHARAFALDIN AL-MUSAWI

**Affiliations:** 1 Department of Molecular Genetics, College of Arts and Sciences, The Ohio State University, Columbus, OH, USA; 2 Cellular and Molecular Research Center, Qom University of Medical Sciences, Qom, Iran; 3 Medical Biotechnology Research Center, AJA University of Medical Sciences, Tehran, Iran; 4 Toxicology Research Center, AJA University of Medical Sciences, Tehran, Iran; 5 Student Research Committee, AJA University of Medical Sciences, Tehran, Iran; 6 Research Fellow School of Life Sciences, University of Sussex, Brighton, UK; 7 Stem Cell Biology Research Center, Yazd Reproductive Sciences Institute, Shahid Sadoughi University of Medical Sciences, Yazd, Iran; 8 The Persian Gulf Marine Biotechnology Research Center, The Persian Gulf Biomedical Sciences Research Institute, Bushehr University of Medical Sciences, Bushehr, Iran; 9 Antimicrobial Resistance Research Center, Mashhad University of Medical Sciences, Mashhad, Iran; 10 Vascular & Endovascular Surgery Research Center, Mashhad University of Medical Sciences, Mashhad, Iran; 11 Department of Mesenchymal Stem Cells, Academic Center for Education, Culture and Research, Qom, Iran; 12 Department of Microbiology, Faculty of Medicine, AJA University of Medical Sciences, Tehran, Iran; 13 College of Food Sciences, Al-Qasim Green University, Babylon, Iraq

**Keywords:** Forkhead Box R2 (FOXR2), Dysregulation, Cancer, Therapeutic, Prognostic

## Abstract

Cancer, a leading cause of global mortality, remains a significant challenge to increasing life expectancy worldwide. Forkhead Box R2 (FOXR2), identified as an oncogene within the FOX gene family, plays a crucial role in developing various endoderm-derived organs. Recent studies have elucidated FOXR2-related pathways and their involvement in both tumor and non-tumor diseases. Dysregulation of FOXR2 has been linked to numerous malignant tumors, spanning the brain, nervous system, thyroid, osteosarcoma, Hodgkin lymphoma, colorectal, liver, pancreatic, lung, breast, ovarian, prostate, female genital tract, endometrial, and uterine cancers. Despite extensive research on FOXR2 dysregulation, its practical applications remain underexplored. This review delves into the mechanisms underlying FOXR2 dysregulation during oncogenesis and its implications for cancer diagnosis, prognosis, and treatment.

## Introduction

Cancer is a major cause of death and a challenge for increasing life expectancy worldwide [1–4] On the other hand, cancer has historically been one of the top causes of mortality globally and the subject of clinical investigations as a significant global public health issue. Recent cancer data show that in the United States alone in 2023, 1,958,310 new instances of cancer were found, and 609,820 cancer patients passed away [5]. Cancer continues to be the biggest obstacle to raising life expectancy in every country in the world, despite notable advancements in its diagnosis and treatment. Cancer patients may not have the best outlook; thus, innovative treatment plans are critical. Molecular targeted treatments offer significant effectiveness with minimal contamination, making them a prominent approach in cancer treatment. Therefore, it is anticipated that patients who are ineligible for drastic surgery may benefit from the understanding of the molecular mechanisms underlying the onset and progression of cancer as well as the identification of novel biomarkers for new targeted treatments.

A transcription factor (TF) is a protein that regulates gene expression by binding to specific DNA sequences near genes, either promoting or inhibiting transcription of the gene into messenger RNA (mRNA), which is then translated into protein. The Forkhead box (FOX) transcription factor family comprises a suite of evolutionarily preserved proteins pivotal in modulating gene expression throughout developmental stages and into maturity. These proteins are increasingly recognized for their roles in the pathogenesis of various human diseases [6]. The FOX gene was first discovered in a mutant form of *Drosophila melanogaster* in the 1980s [7]. The FOX gene subgroup regulates many complex molecular pathways that are disrupted in various cancers [6,8,9]. The FOX family genes play a crucial role in the onset of a myriad of diseases, including diabetes mellitus, congenital anomalies, and cancer. This gene family boasts at least 43 human variants, categorized into several subfamilies. Among these is the FOXR subgroup, which includes Forkhead Box R1 (FOXR1) and Forkhead Box R2 (FOXR2), both of which are significant in their respective regulatory functions.

FOXR2, designated as Forkhead Box N6 (FOXN6), is a transcription factor in humans that emerged from bioinformatic analyses in 2004 [10]. Characterized by a forkhead domain located at its C-terminal region, it exhibits a high degree of conservation with other FOX family proteins. Notably, FOXR2 shares 57.7% of its genetic makeup with FOXR1, its counterpart within the FOXR subgroup. Functionally, FOXR2 is known to parallel the activity of MYC, a well-known oncogene, thereby facilitating cellular proliferation [11]. FOXR2 is a testis-specific gene on the X chromosome that belongs to the FOX gene family. It has been found to play a key role in the development and progression of various cancers and in regulating some cellular functions. FOXR2 is also involved in human central nervous system (CNS) tumors. Previous large-scale transposon mutagenesis screenings have suggested that FOXR2 is a potential tumor driver gene in medulloblastoma and malignant peripheral nerve sheath tumors [12,13].

Tsai’s team’s research uncovered that FOXR2 is implicated in numerous cancers, including glioma, osteosarcoma, melanoma, and lung cancer. Their pan-cancer study across thousands of samples revealed FOXR2 overexpression in most cancers due to a novel, hypomethylated promoter essential for cancer cell proliferation. Additionally, FOXR2 activates the ETS transcriptional pathways, contributing to tumor development, and influences MAPK signaling, underscoring its role as a widespread oncogene [14]. In this review, we summarize the recent findings on the roles and mechanisms of FOXR2 in cancer and highlight its clinical significance as a biomarker and a target for cancer treatment. In this article, we review the aberrant expression, molecular mechanism, and clinical significance of FOXR2 in cancer.

## Search Strategy and Study Selection Process

We searched PubMed (https://pubmed.ncbi.nlm.nih.gov/), Web of Science (https://www.webofscience.com), Scopus (https://www.scopus.com), Embase (https://www.elsevier.com/products/embase) (accessed on 19 January 2024) ProQuest (https://www.proquest.com), and The Cochrane Library (https://www.cochrane.org/) for articles published until August 2024. We used a combination of Mesh and free keywords such as FOXR2, cancer, and therapeutic as part of the search strategy.

## Function, Structure, Localization, and Expression of FOXR2

**Figure 1:**
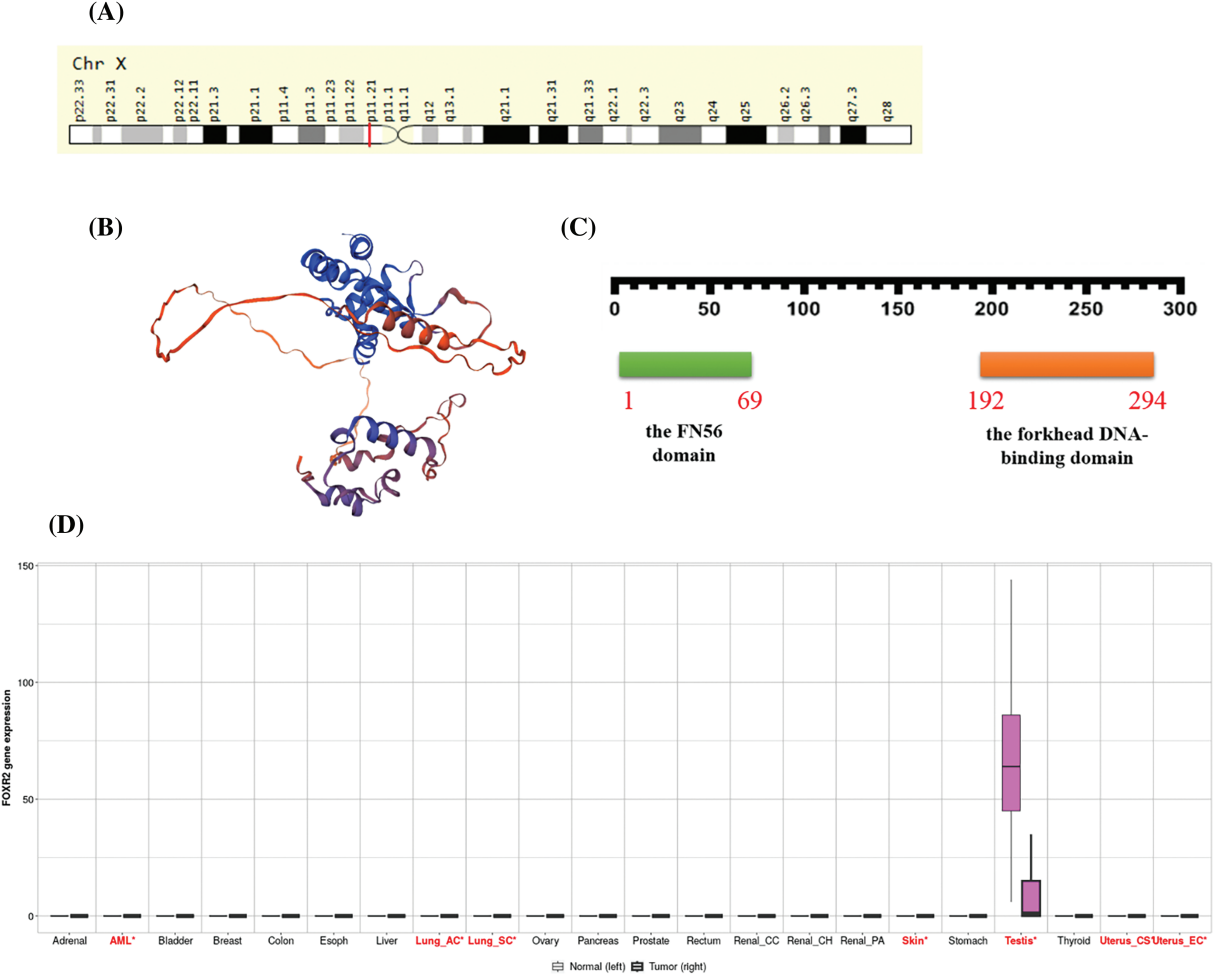
Functional roles of FOXR2 across multiple cancer types. (A) FOXR2 Gene in genomic location: bands according to Ensembl, locations according to GeneLoc. (B) Structure prediction of FOXR2 from Alphafold project, Version 2. (C) The sequence of amino acids in FOXR2, including the forkhead DNA-binding domain (from amino acid 192 to amino acid 294) and the FN56 domain (from amino acid 1 to amino acid 69). (D) Pan-cancer analysis of FOXR2 gene functions in The Cancer Genome Atlas (TCGA) cohort.

The forkhead box or winged helix domain, a characteristic DNA-binding domain present in members of the FOX family of proteins, has been preserved throughout the development of TFs. They have evolved through extensive diversification and specialization in different organisms [6]. FOX proteins have various roles in maintaining cellular homeostasis and their expression is tightly controlled in adult tissues. One of the FOX proteins, FOXR2, has been identified as an oncogene, a gene that can cause cancer when mutated or overexpressed, which promotes the development of some cancers [7] FOXR2 is located on the X chromosome at Xp11.21 (Fig. 1A) and encodes a TF that is normally expressed only in the testis (Fig. 1B).

FOXR2 has been implicated as an oncogene in some types of malignancies. However, the oncogenic significance of FOXR2 in all malignancies is not fully understood, nor are the mechanisms by which it promotes tumor growth. FOXR2 has one transcript, which means that it produces one type of mRNA that can be translated into protein (http://nov2020.archive.ensembl.org/Homo_sapiens/Gene/Summary?db=core;g=ENSG00000189299;r=X:55623400-55626192;t=ENST00000339140) (accessed on 26 January 2024). FOXR2 expression is seen in several common malignancies, including neuroblastoma, glioma, sarcoma, and osteosarcoma [14].

The FN56 domain is a feature shared by FOXR2 and its related proteins in other animals [10]. Katoh et al. also found FOXR2 proteins in mice and rats, which had about 54% and 52% of the same amino acids as human FOXR2, respectively [10]. Based on information from The Human Protein Atlas (https://www.proteinatlas.org/ENSG00000189299-FOXR2/subcellular) (accessed on 26 January 2024), FOXR2 is localized to the nucleoplasm, which is the part of the nucleus where DNA and other proteins are found. This makes sense because TFs need to bind to DNA to regulate gene expression. FOXR2 may interact with other proteins in the nucleoplasm to form complexes that control the transcription of specific genes. According to Expasy, FOXR2 has 311 amino acids in its sequence. The sequence of amino acids in FOXR2 is unique to this protein, and it contains the forkhead DNA-binding domain (from amino acid 192 to amino acid 294) [14] and the FN56 domain (from amino acid 1 to amino acid 69), as illustrated in Fig. 1C [10]. All FOX proteins share the forkhead DNA-binding domain, enabling them to identify and bind specific DNA sequences, thereby regulating gene transcription via RNA polymerase II (Fig. 1D). The FN56 domain, unique to FOXR2 and its orthologs, is thought to influence protein interactions or stability [14].

**Figure 2:**
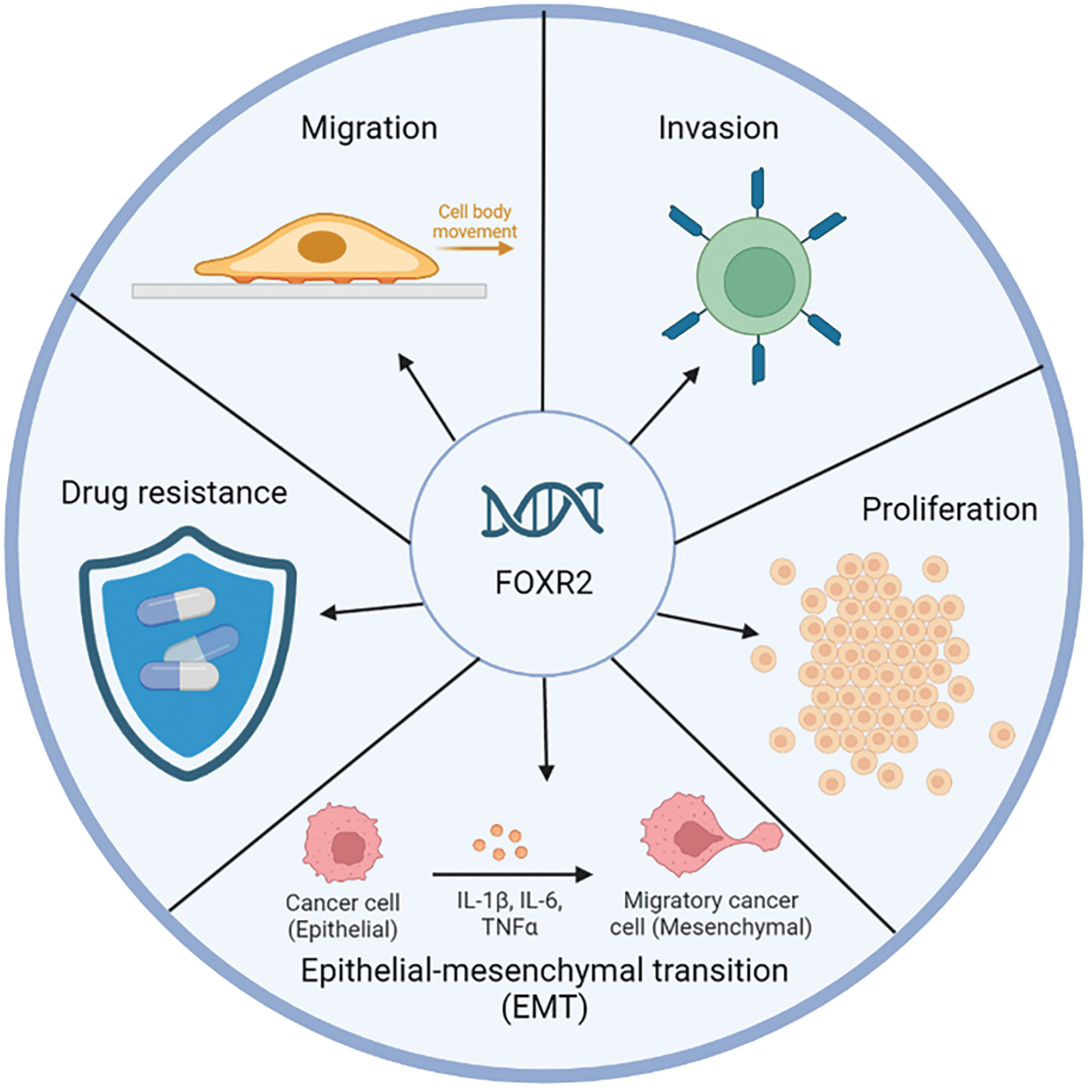
Biological function FOXR2 in cancer. FOXR2 is a critical oncogene involved in the proliferation and malignancy of various cancers. Its unique expression in cancers and interaction with key transcription factors make it a valuable target for future cancer therapies.

FOXR2 dysregulation is involved in many aspects of cancer progression, such as altering oncogenic pathways, escaping apoptosis, enhancing drug resistance, inducing autophagy, facilitating epithelial to mesenchymal transition, improving DNA repair, and modifying cancer stem cell properties (Fig. 2). FOXR2 also has potential as a diagnostic and prognostic biomarker and a therapeutic target for cancer patients.

## FOXR2 Network

**Figure 3:**
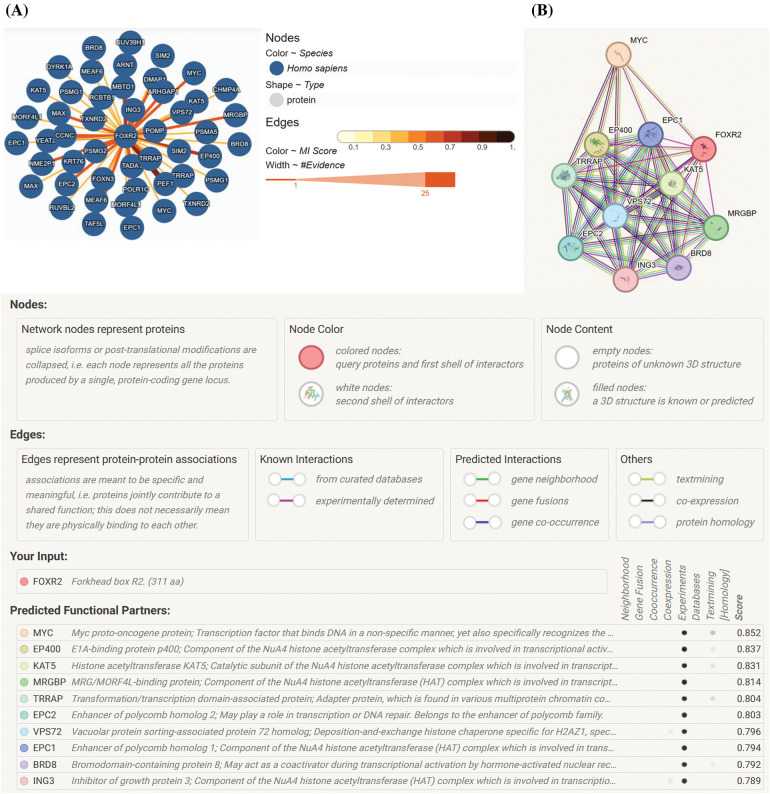
FOXR2 network. (A) The FOXR2 network’s physical protein-protein interactions, produced by STRING (URL: https://string-db.org/) [16]. (B) A network of FOXR2 that was adapted from the IntAct database (URL: https://www.ebi.ac.uk/intact/home) (accessed on 26 January 2024) [17].

Research findings indicate that FOXR2 interacts with various proteins, forming networks depicted in models generated by online tools such as those available in the STRING and IntAct databases [11]. Specifically, Fig. 3A,B illustrates the protein-protein interaction structure within the FOXR2 network (URL: https://string-db.org/, accessed on 26 January 2024). Some members of this network may belong to the histone acetyltransferase (HAT) complex, including KAT2A, KAT2B, KAT5, EP400, and TRRAP. Notably, interactions between MYC and FOXR2 are implicated in transcriptional control and carcinogenesis. While the literature mentions TRRAP’s connection with FOXO3 only once, models suggest a TRRAP-FOXR2 connection. TRRAP plays a crucial role in various chromatin complexes, including HATs, and is essential for transcriptional activation mediated by p53/TP53, E2F1, and E2F4 [15]. Additionally, key HATs involved in transcriptional activation include KAT2A, KAT2B, YEATS4, ING3, and EP400. VPS72 is implicated in histone chaperone activity for H2AZ1, facilitating its deposition into nucleosomes and thus controlling chromatin remodeling and gene transcription. TAF12 and TAF6L are also important regulators, with TAF6L involved in somatic reprogramming and the MYC regulatory network, orchestrating gene expression in embryonic stem cells through H3K9ac deposition and MYC recruitment. The FOXR2 network is intricately linked to tumor development and progression through its roles in promoting cell proliferation, inhibiting apoptosis, enhancing metastasis, and interacting with key oncogenic pathways [15].

## Molecular Mechanisms of FOXR2

Despite the fact that structural variations have been found to activate FOXR2 in some cancers, such as peripheral neuroblastomas [18] and CNS tumors [19].

**Figure 4:**
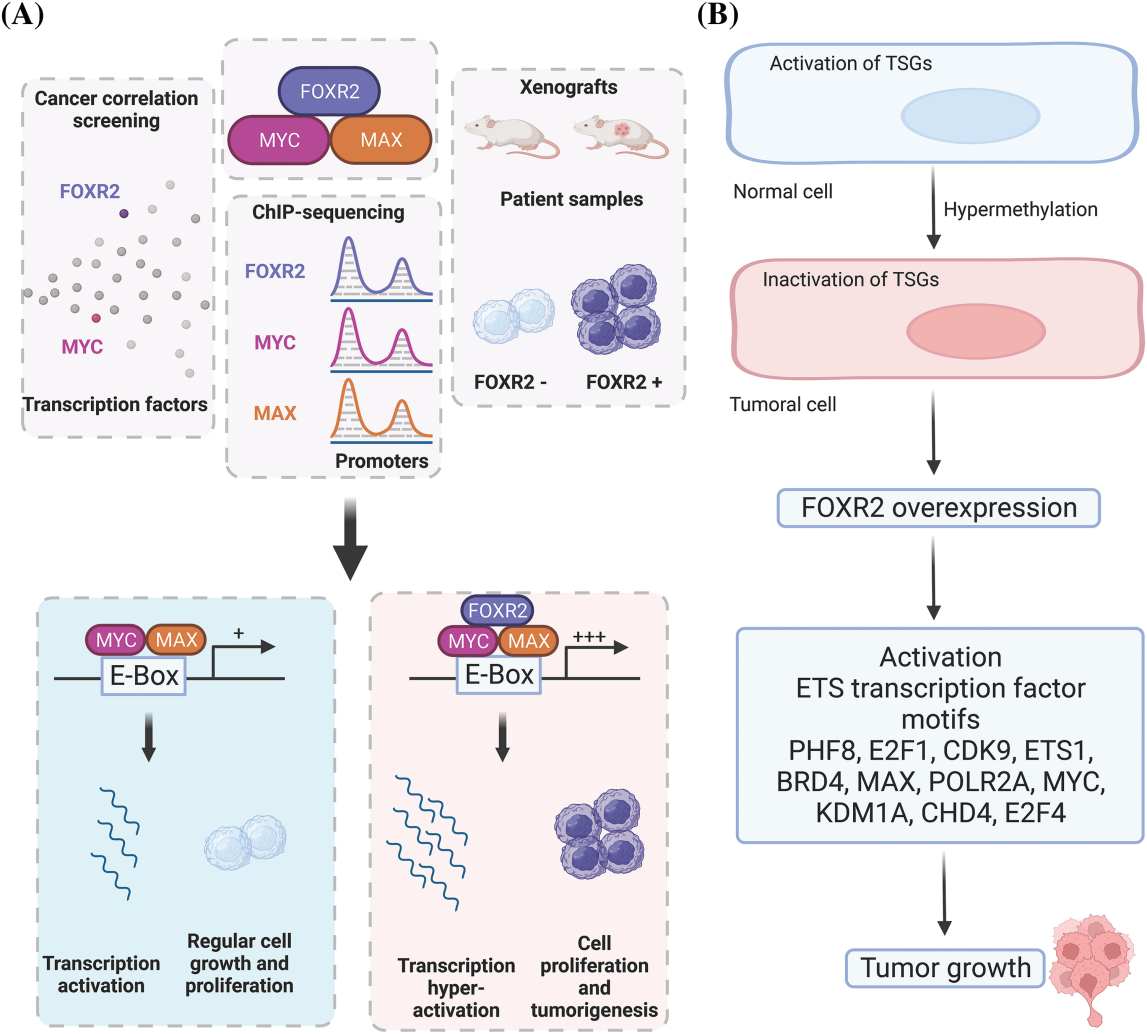
Molecular mechanisms of FOXR2 in cancer. (A) FOXR2 interacts with MYC and MAX in oncogenesis. (B) Overexpression of FOXR2 activates EST TFs that induce tumor growth. FOXR2 acts as an oncogene by upregulating genes that enhance cell proliferation and downregulating apoptotic genes, interacts with key signaling pathways like MYC/MAX to support tumor growth, modifies the epigenetic landscape by influencing chromatin structure and histone modifications, and alters the tumor microenvironment to favor angiogenesis and immune evasion.

Moreover, the normal distribution and function of FOXR2 in different tissues are unclear. The mechanisms by which FOXR2 promotes tumor growth are not well understood. Previous studies have mainly focused on how FOXR2 stabilizes MYC isoforms [18], which are proteins that regulate cell growth and survival [14]. However, it is possible that FOXR2 interacts with other proteins or molecules that contribute to its oncogenic activity. TFs often work together to influence gene expression by forming transcriptional complexes. For example, other FOX proteins have been shown to interact with other TFs or bind to different DNA motifs to mediate their effects [18,20]. It is still unknown if FOXR2 also uses similar mechanisms to promote oncogenesis. Furthermore, it was demonstrated that 8% of tumors, or more than 70% of all cancer lineages, showed signs of FOXR2 expression [18]. Different FOXR2 activation mechanisms were also uncovered, including a unique nonstructural variation mechanism that accounted for the bulk of FOXR2-expressing malignancies. Notably, they revealed a relationship between FOXR2 and the activation of TF circuits unique to ETS and uncovered that ETS TFs are necessary for FOXR2-mediated transformation (Fig. 4A,B). Consequently, the discovery of FOXR2’s hijacking of ETS transcription circuits shows how TF families collaborate to promote cancer and expands the methods known to activate ETS TFs [18].

Medulloblastoma and CNS neuroblastoma are two types of tumors that are typically synaptophysin-positive. Unlike many other tumors, they often do not express glial fibrillary acidic protein (GFAP) or vimentin. A notable characteristic of these cancers is the overexpression of Thyroid Transcription Factor 1 (TTF-1), which could serve as a useful immunomarker. Additionally, these malignancies frequently exhibit chromosomal rearrangements involving the FOXR2 gene, leading to increased FOXR2 expression, a genetic signature common to these types of cancers [21].

Hence, Analysis of frequent copy number changes (breakpoints of the FOXR2 locus on Xp11.21, gain of 1q), structural FOXR2 rearrangements, and next-generation sequencing, and DNA methylation classification are the most straightforward ways to obtain molecular confirmation of these tumors [21].

FOXR2 is also a gene in medulloblastoma that has undergone the highest insertional mutagenesis using the Sleeping Beauty (SB) transposon. A tiny subset of the sonic hedgehog (SHH) subgroup of medulloblastoma, known as FOXR2, is highly expressed. According to Li and colleagues, this gene contributes to the development of CNS-embryonal malignancies by working with the MYC/MYC-associated factor X (MAX) complex and facilitating the growth of cancer cells, as well as by overexpressing in the presence of a Trp53 deficiency [22] (Fig. 4A). In addition to prostate, liver, and breast cancers, this protein shows oncogenic activity in a variety of cancer cell types, including malignant peripheral nerve sheath tumors. Importantly, CNS-embryonal tumors (ET) developed with 100% penetrance when FOXR2 overexpression was combined with a Trp53 deficiency [23].

Additionally, Pineoblastoma-FOXR2/MYC and CNS-NB-FOXR2 represent distinct molecular subgroups within the broader category of pediatric brain tumors, each with unique genetic characteristics and implications for treatment and prognosis. Pineoblastoma is a rare, aggressive type of brain tumor that originates in the pineal gland. The FOXR2/MYC subgroup indicates tumors that have genetic alterations involving the FOXR2 and MYC genes. These genetic changes can drive tumor growth and may influence the tumor’s response to treatment. Understanding the role of FOXR2/MYC in pineoblastoma can help in developing targeted therapies aimed at these specific genetic alterations. On the other hand, CNS Neuroblastoma with FOXR2 activation (CNS-NB-FOXR2) is another subgroup within pediatric brain tumors, characterized by alterations in the FOXR2 gene [24–27]. These tumors share some similarities with neuroblastomas, which are typically found outside the CNS, but CNS-NB-FOXR2 occurs within the CNS. The identification of FOXR2 as a key player in these tumors suggests potential targets for therapy and highlights the importance of genetic profiling in optimizing treatment strategies. Both subgroups underscore the significance of molecular diagnostics in understanding and treating pediatric brain tumors. By identifying specific genetic alterations, clinicians can tailor treatment approaches to the individual tumor’s characteristics, potentially improving outcomes for affected children.

## FOXR2-Associated Tumorigenesis and Regulation

FOXR2-associated tumorigenesis involves the overexpression of the FOXR2 gene in various cancers, including gliomas, lymphomas, and prostate cancer. This overexpression leads to increased cell proliferation, inhibition of apoptosis, and enhanced metastatic potential. FOXR2 exerts its oncogenic effects by regulating the expression of genes involved in critical cellular processes. As a transcription factor, FOXR2 influences genes that control the cell cycle, apoptosis, and metastasis, thereby promoting tumor growth and progression [14,28].

One of the key mechanisms by which FOXR2 promotes tumorigenesis is through its interaction with other oncogenic pathways. For instance, FOXR2 can enhance the activity of the Wnt/β-catenin pathway, which is known for its role in cell proliferation and survival. Additionally, FOXR2 contributes to the epithelial-mesenchymal transition (EMT), a process essential for cancer metastasis. EMT enables cancer cells to acquire migratory and invasive properties, facilitating their spread to distant organs [29].

The regulation of FOXR2 is complex and involves multiple layers of control. At the transcriptional level, FOXR2 expression can be modulated by other signaling pathways and transcription factors that either promote or suppress its transcription. Post-translational modifications, such as phosphorylation, can also impact FOXR2’s stability and activity, further influencing its function in cancer cells. Moreover, microRNAs (miRNAs) can regulate FOXR2 expression post-transcriptionally by targeting its mRNA, adding another layer of control over its activity [11].

In summary, FOXR2 plays a significant role in tumorigenesis by regulating key genes and interacting with crucial oncogenic pathways. Its overexpression leads to increased cell proliferation, survival, and metastatic potential, making FOXR2 a potential target for cancer therapy. Understanding the regulatory mechanisms of FOXR2 could provide new insights into cancer biology and aid in the development of targeted treatments.

## Role of FOXR2 on Several Cancers

Numerous cancers, including pediatric high-grade gliomas and diffuse midline gliomas, thyroid cancer, Hodgkin lymphoma, and osteosarcoma, were found to have FOXR2 upregulated (Table 1).

**Table 1:** Various aspects of FOXR2 across different cancer types

Cancer type	Assessed cell lines	Interacting genes and proteins	Novel Therapeutic/Diagnostic	Expression	Function	Year	Reference
Colorectal	SW480, HT29, and LOVO	Shh, Gli1, and Ptch1	Potential therapeutic target	↑	Promotes proliferation, invasion, EMT	2017	[30]
Hepatocellular	Huh7, YY-8103, L02, Hep3B, HCC-LM3, and WRL68	β-catenin, Skp2, c-Myc, and Gli-1	Potential therapeutic target	↑	Contributes to cell proliferation and malignancy	2016	[31]
Non-Small Cell Lung Cancer (NSCLC)	A549, H157, and BEAS-2B and NSCLC tissues and adjacent normal tissues	Components of the Wnt/β-catenin signaling pathway, β-catenin, cyclinD1 and c-Myc	A novel therapeutic target	↑	Promotes cell proliferation and invasion	2018	[32]
Lung Adenocarcinoma (LAC)	HEB, A549, H1299, H1975 and LAC tissues and adjacent normal tissues	PJA1	A novel therapeutic target	↑	Influences apoptosis and invasion in lung adenocarcinoma cells	2021	[33]
NSCLC	NSCLC tissues and adjacent normal tissues	circ-PGC and miR-532-3p	A novel therapeutic target	↑	Promoting NSCLC development and progression	2021	[34]
Breast	–	–	Diagnostic marker	↑	Associated with poor prognosis	2016	[35]
Ovarian	SK-OV-3, CaoV-3 OV-1063, SV40 CoC1, and OVCAR3	Sonic hedgehog	Potential therapeutic target	↑	Promotes metastasis and growth in ovarian cancer by stimulating angiogenesis and activating the Hedgehog signaling pathway	2018	[36]
Ovarian	PTX-sensitive ovarian cancer tissues and PTX-resistant ovarian cancer tissues	circCELSR1, miR-1252	A novel therapeutic target	↑	Promotes paclitaxel resistance, metastasis, angiogenesis, activates Hedgehog signaling	2019	[37]
Ovarian	Ovarian cancer cell lines	circANKRD17 and FUS	A novel therapeutic target	↑	Involved in paclitaxel resistance in ovarian cancer cells	2023	[38]
Prostate	PC3, DU145	β-catenin, cyclinD1 and c-Myc	Potential therapeutic target	↓	Suppresses tumorigenesis, growth, metastasis	2017	[39]
Endometrial adenocarcinoma	HEC-1A, Ishikawa	miR-202	Diagnostic marker	↑	Promotes cell proliferation	2017	[40]
Central Nervous System (CNS) Embryonal tumors	Various CNS embryonal tumor cell lines	Trp53	Novel therapeutic marker	↑	Promoting the formation of CNS-embryonal tumors, especially when Trp53 is deficient	2019	[22]
Medulloblastoma	C17.2	HIF1A, IFG1, TP53, ERRB and MYC	Potential therapeutic target	↑	Regulates genes promoting tumor growth and survival	2015	[41]
Medulloblastoma	SHH subtype of human medulloblastoma	Tgif2, and Alx4	Potential novel therapeutic target and diagnostic marker	↑	Drive cell proliferation, enhance survival, and contribute to the aggressive nature of medulloblastoma	2014	[13]
Pediatric brain cancer	Various medulloblastoma cell lines	MYCN and MYC	Potential as both a therapeutic target and a diagnostic marker	↑	Promoting cell division, growth, and survival	2020	[42]
Embryonal Brain Tumors (including medulloblastoma and other similar pediatric brain cancers)	Various embryonal brain tumor cell lines, including medulloblastoma and Trp53-deficient mice	Nestin, Tuj1, synaptophysin and NeuN, and Olig2	A promising candidate for novel therapeutic interventions and diagnostic tools	↑	Promotes tumor growth by enhancing cell proliferation, survival, and resistance to apoptosis, contributing to the malignancy of embryonal brain tumors	2019	[43]
Central Nervous System (CNS) Embryonal tumors (including Medulloblastoma)	Genetically engineered mouse models and HEK293 and various embryonal brain tumor cell lines, including medulloblastoma	N-MYC and C-MYC	A novel therapeutic target and a potential diagnostic marker	↑	Promoting tumor growth, cell proliferation, and survival	2019	[44]
Pan-Cancer (including multiple types of cancers, not limited to a specific kind)	A variety of cancer cell lines from different types of cancers	ETS transcriptional circuits	A novel therapeutic target and potential diagnostic marker across multiple cancer types	↑	Promoting oncogenic processes such as cell proliferation, survival, and metastasis	2022	[14]
CNS-PNETs (Central Nervous System Primitive Neuroectodermal Tumors)	Various CNS-PNET cell lines and patient-derived tumor samples	Genes and proteins involved in neural development, cell proliferation, and tumor progression	A novel therapeutic target and a diagnostic marker for CNS-PNETs	↑	Promotes oncogenic processes such as uncontrolled cell growth and survival, contributing to the aggressiveness of these tumors	2016	[19]
Central Nervous System (CNS) Neuroblastic tumor	Case-specific tumor samples from a patient with a CNS neuroblastic tumor	Synaptophysin and GFAP	A potential therapeutic target and diagnostic marker	↑	Promotes both neuronal and glial differentiation within the tumor	2020	[45,46]
Rare CNS Embryonal Tumor Entities (including medulloblastoma and other similar pediatric brain cancers)	Patient samples from rare CNS embryonal tumor entities	Genes and proteins relevant to CNS embryonal tumor biology	A potential therapeutic target and diagnostic marker	↑	Plays a role in the pathogenesis of rare CNS embryonal tumor entities	2021	[23]

Note: ↑, upregulated, ↓, downregulated.

**Figure 5:**
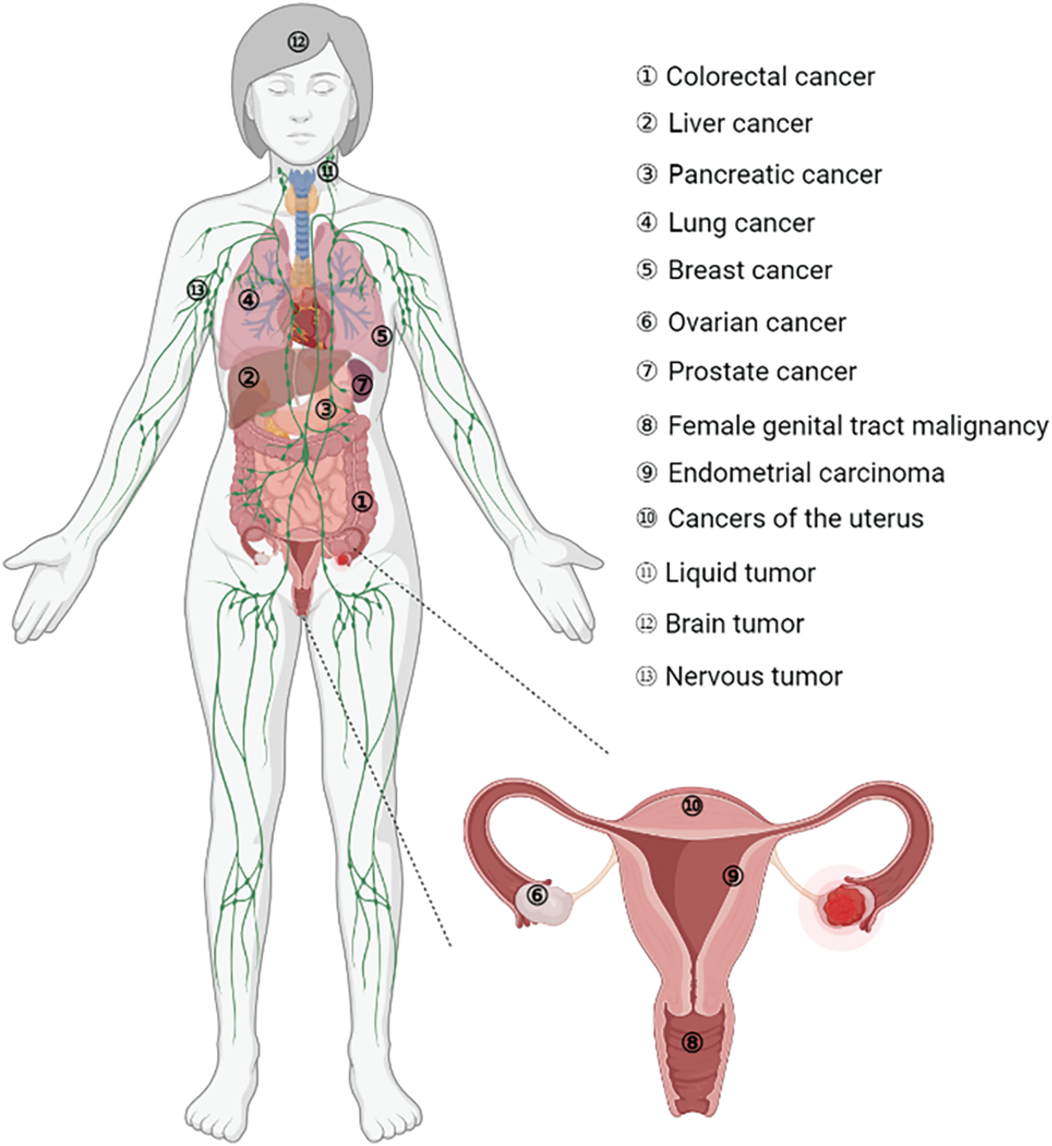
Involvement of FOXR2 in different cancers.

These cancers also included colorectal, liver, pancreatic, lung, breast, ovarian, and prostate cancers, as well as endometrial carcinoma and central nervous system-embryonal tumors (Fig. 5). FOXR2 dysregulation in cancer involves several critical mechanisms. One of the primary factors is the overexpression of FOXR2, which is commonly observed in various cancers, including gliomas, lymphomas, and prostate cancer. This overexpression is often correlated with poor prognosis and increased tumor aggressiveness. The elevated levels of FOXR2 protein contribute to the malignant behavior of cancer cells, promoting uncontrolled proliferation and survival.

Gene amplification is another significant aspect of FOXR2 dysregulation. The FOXR2 gene can undergo amplification, leading to an increased dosage of the gene product. This amplification enhances the oncogenic potential of FOXR2, further driving tumor growth and progression. Additionally, dysregulation at the transcriptional level can occur due to mutations or abnormal activity of transcription factors that regulate FOXR2 expression. Such transcriptional dysregulation results in elevated or inappropriate expression of FOXR2 in cancer cells.

Post-translational modifications play a crucial role in the regulation of FOXR2. Changes in these modifications, such as phosphorylation, can impact the stability, localization, and activity of the FOXR2 protein, contributing to its dysregulation in cancer. Furthermore, alterations in the levels or activity of specific microRNAs (miRNAs) that target FOXR2 mRNA can lead to its overexpression. Reduced levels of these miRNAs result in increased production of the FOXR2 protein, enhancing its oncogenic effects.

Epigenetic changes are also involved in FOXR2 dysregulation. Modifications such as DNA methylation and histone modifications can influence FOXR2 expression. Abnormal epigenetic regulation can lead to the overexpression of FOXR2 in cancer cells, contributing to tumorigenesis. Moreover, FOXR2 can interact with and enhance the activity of other oncogenic signaling pathways, such as the Wnt/β-catenin pathway. This crosstalk amplifies the oncogenic effects of FOXR2 dysregulation, promoting cancer progression.

Finally, somatic mutations in the FOXR2 gene can lead to changes in its function or expression levels, further contributing to cancer development and progression. These mutations can result in the gain of function or loss of regulatory control, making FOXR2 a potent driver of tumorigenesis. Understanding these key points of FOXR2 dysregulation is crucial for identifying potential therapeutic targets and developing strategies to counteract its oncogenic effects in cancer.

### Digestive system

#### Colorectal cancer

With regard to FOXR2 involvement, various malignancies are categorized (Fig. 5). The role of FOXR2 in colorectal cancer (CRC) is a common and deadly disease. It is the second greatest cause of cancer death globally and the second most common cancer diagnosis [30]. In 2022, more than 160,000 new cases are expected in the US alone, with half of them occurring in patients under 66 years old [30]. Experimental studies have shown that FOXR2 was overexpressed in CRC tissues and cells *in vitro* [30,47]. Previous studies have shown that FOXR2, a gene involved in CRC, regulates several aspects of CRC cell behavior, such as invasion, proliferation, and the ability to switch between epithelial and mesenchymal states (EMT) [47]. Moreover, FOXR2 affects the expression of key components of the Hedgehog signaling pathway, such as SHH, Gli1, and Ptch1, in SW480 cells, a CRC cell line. These findings imply that FOXR2 is critical for promoting CRC cell growth, metastasis, and EMT [47].

#### Liver cancer

According to the Cancer Stat Facts website, the estimated new cases of intrahepatic bile duct and liver cancer in the United States for 2023 are 41,210, with an estimated 29,380 deaths. Incidence rates of liver cancer have more than quadrupled since 1980, while mortality rates have more than doubled. The American Cancer Society website notes that liver cancer is more prevalent in Southeast Asian and sub-Saharan African nations compared to the US, being the most prevalent type of cancer in many of these regions. Globally, liver cancer affects over 800,000 people annually, with more than 700,000 deaths, contributing significantly to cancer mortality worldwide [5] Wang et al. [31] demonstrated that FOXR2 upregulation enhanced the growth of tumor xenografts in nude mice. They also identified Skp2, β-catenin, Gli-1, and c-Myc as potential downstream targets of FOXR2 that mediate its effects on cell proliferation and malignancy, using quantitative real-time PCR analysis. Further *in vitro* and preclinical studies are needed to elucidate the function and mechanism of FOXR2 in liver cancer.

#### Pancreatic cancer

According to the Cancer Stat Facts website (https://seer.cancer.gov/statfacts/html/pancreas.html) (accessed on 19 January 2024), the estimated number of new cases of pancreatic cancer in the United States for 2022 is 62,210 and the estimated deaths are 49,830 [48]. These statistics are based on 2016–2020 cases and fatalities and are age-adjusted. The website also shows that both the rate of new cases and deaths rose between 2010 and 2019. In 2024, it is estimated that there will be 66,440 new cases of pancreatic cancer, representing approximately 3.3% of all new cancer diagnoses. Furthermore, the anticipated number of deaths from pancreatic cancer is 51,750, accounting for 8.5% of all cancer-related fatalities, highlighting the critical impact of this disease, based on the Cancer Stat Facts website. Tsai et al. [14] reported that FOXR2 expression was altered in pancreatic adenocarcinoma. Furthermore, functional tests demonstrated that FOXA2 inhibits the colony formation, growth, migration, and invasion of pancreatic cancer cells, demonstrating a tumor suppressor effect [49]. However, the function and mechanism of FOXR2 in pancreatic cancer remain largely unknown and require further experimental investigation.

### Lung cancer

Wang et al. [32] investigated the role of FOXR2 in non-small cell lung cancer (NSCLC). They found that down-regulating FOXR2 reduced the invasion and proliferation of NSCLC cells *in vitro* and the metastasis and growth of NSCLC cells *in vivo*. They also found that down-regulating FOXR2 inhibited the Wnt/β-catenin pathway and decreased the expression of c-Myc, cyclinD1, and β–catenin in NSCLC cells [32].

Luo et al. [33] revealed that the E3 ligase PJA1 regulates the stability of FOXR2 by targeting it for ubiquitin-mediated degradation and predicts a favorable prognosis for lung adenocarcinoma (LAC) patients. They also showed that the degradation of FOXR2 by PJA1 affects LAC apoptosis and invasion. In the study by Xia et al. [34], it was demonstrated that overexpression of FOXR2 and circ-PGC in NSCLC tissues and cells could be linked to the disease’s progression. The research revealed that reducing circ-PGC levels led to a decrease in NSCLC cell growth, clonogenicity, movement, invasiveness, and glycolytic activity. Conversely, these suppressed cellular functions were restored by increasing FOXR2 levels. Additionally, circ-PGC was shown to elevate FOXR2 expression by outcompeting miR-532-3p for a mutual binding site. Furthermore, the suppression of circ-PGC resulted in lower levels of β-catenin and c-Myc, which could be reversed by either inhibiting miR-532-3p or overexpressing FOXR2. The findings suggest that the circ-PGC/miR-532-3p/FOXR2 regulatory axis may activate the Wnt/β-catenin signaling pathway, enhancing FOXR2 expression and thus contributing to the advancement of NSCLC [34]. By reducing the expression of circABCB10, a circular RNA, the migration and proliferation of NSCLC cells can be inhibited [50]. This is because circABCB10 acts as a molecular sponge for miR-1252, which normally targets and suppresses FOXR2. Fluorescein reporting assays confirmed that circABCB10 increased FOXR2 levels by sequestering miR-1252, while animal studies showed that knocking down circABCB10 slowed down tumor growth [50]. The authors of the study hypothesized that the biomarker FOXR2 may be used to diagnose and predict lung cancer and serve as a possible treatment target [32–34], which is an exciting field to explore.

### Breast cancer

Breast cancer is the most common cancer worldwide [51]. More than 1.7 million new cases and more than 521,900 deaths from breast cancer occur in women each year, making it the most prevalent and lethal disease in this group of patients [35,51]. Breast cancer cell lines and primary breast tumors were both shown to overexpress FOXR2 [10]. Song et al. investigated the potential of FOXR2 as a prognostic and predictive biomarker in breast cancer by measuring its expression using real-time PCR and immunohistochemistry (IHC) staining [35]. They found that both FOXR2 protein and mRNA levels were higher in breast cancer samples than in normal breast tissues. Furthermore, Yan et al. showed that in triple negative breast cancer with paclitaxel resistance, a number of genes, including FOXR2, function as negative moderators of cancer stemness [52].

Immunohistochemistry analysis indicated a clear link between the level of FOXR2 and key markers of tumor aggressiveness, such as size and proliferation rate. Elevated levels of FOXR2 correlated with reduced survival rates, particularly in cases involving smaller tumors and the presence of lymph node metastases. Furthermore, statistical analysis confirmed FOXR2 as a standalone prognostic indicator for breast cancer. These findings point to FOXR2’s potential role as an important biomarker in identifying and predicting the course of breast cancer [35].

### Genital system

#### Ovarian cancer

According to the American Cancer Society, ovarian cancer will affect 19,880 women and cause 12,810 deaths in the United States in 2022 [53–55]. The five-year survival rate for ovarian cancer is only 20%, as most cases are detected at a late stage [56]. There is a need to enhance the current methods of diagnosis and prognosis of ovarian cancer [53,57]. Overexpression of FOXR2 is linked to metastasis and tumor progression, particularly in ovarian cancer, where it correlates with worse histologic grade and poor survival [36,58]. Zhang et al. identified FOXR2 as a novel target of miR-1252 [37]. They found that circCELSR1, a circular RNA, increased FOXR2 expression by sequestering miR-1252 in Paclitaxel-resistant ovarian cancer cells. Additionally, circANKRD17 (also known as circ 0007883), another circular RNA, confers paclitaxel resistance in ovarian cancer by binding to FUS, an RNA-binding protein, and stabilizing FOXR2 [38]. The research findings reveal a complex interplay where FOXR2 not only governs the carcinogenic traits and epithelial-mesenchymal transition (EMT) in ovarian cancer (OC) cells but also enhances the Hedgehog signaling pathway. Intriguingly, this pathway, in turn, regulates the activity of FOXR2. This reciprocal relationship suggests that FOXR2 is both a regulator and a target of the Hedgehog signaling, which is known to drive angiogenesis, contributing to the metastasis and advancement of OC [36].

#### Prostate cancer

With an expected 268,490 new cases and 34,500 fatalities from the disease globally in 2022, prostate cancer is the second leading cause of death and the fifth most lethal cancer in males [48]. Prostate cancer is the most frequently diagnosed cancer in 112 countries and the most lethal cancer in 48 countries. It should be noted that the incidence of prostate cancer is expected to increase due to population aging and economic growth [59]. In 2024, it’s estimated that there will be 299,010 new cases of prostate cancer in the United States. Approximately 35,250 deaths are expected from prostate cancer. The lifetime probability of developing prostate cancer for men is 12.9% [60]. Xu et al. [39] showed that FOXR2 is essential for cell proliferation and invasion during the progression of prostate cancer, partly by inhibiting the Wnt/β-catenin signaling pathway. Therefore, the therapeutic target FOXR2 may have promise for the treatment of prostate cancer [39]. However, more research is needed to elucidate the role and exact mechanism of FOXR2 in prostate cancer.

#### Endometrial carcinoma

The most typical malignancy of the female genital system is endometrial carcinoma (EC). Common signs include abnormal uterine bleeding, pelvic pain, and uterine enlargement. EC is the most common gynecologic malignancy in the U.S., with over 66,000 new cases expected in 2023 [61]. In a study by Deng et al. the amount of FOXR2 protein was assessed using Western blot and immunohistochemistry in 90 endometrioid adenocarcinomas (EAC) tissues and 40 matched normal tissues. The study also analyzed the survival of EAC patients in relation to FOXR2 expression. The study revealed that FOXR2 was significantly increased in EAC tissues and that this overexpression was correlated with a poor prognosis [40].

Moreover, Deng et al. demonstrated that miR-202 suppresses cell proliferation in endometrial adenocarcinoma by targeting FOXR2 [40]. Thus, FOXR2 may be a negative prognostic factor and a therapeutic target for EAC. However, more research is needed to elucidate the role and exact mechanism of FOXR2 in EAC.

In addition to endometrial carcinoma, FOXR2 is implicated in various malignant tumors, notably uterine cancers like endometrial and cervical carcinomas. High FOXR2 mRNA levels in serum correlate with increased expression of cancer markers CA199, SCCA, CA125, and CEA, and are associated with poor clinical outcomes and prognosis in uterine malignancies. Together, FOXR2 and 3D-PDU present a potential method for detecting these cancers [62].

### Brain & other nervous system

#### Central/peripheral nervous system-embryonal tumors

Children are susceptible to a class of extremely aggressive cancers known as CNS embryonal tumors [63]. The histological similarity of these tumors hindered their accurate diagnosis and the development of effective treatment strategies until recently [63]. The study by Bielamowicz et al. presents two cases of pediatric CNS embryonal tumors initially diagnosed with EWSR1-PLAGL1 rearrangements, which at relapse were reclassified as INI-1 deficient tumors due to the acquisition of SMARCB1 alterations and loss of INI-1 expression [64]. Rahrmann et al. demonstrated that FOXR2, a proto-oncogene, has a novel role in promoting anchorage-independent growth and tumorigenicity in human malignant peripheral nerve sheath tumors [not central tumor] [12]. FOXR2 stimulated tumor growth in the olfactory bulb (OB) and brainstem (BS) [65]. However, FOXR2 only enhanced the progression of CNS-ET when the Trp53 gene was mutated [22]. In summary, FOXR2, identified as an oncogene within the FOX gene family, plays a crucial role in developing various endoderm-derived organs and has been linked to numerous malignant tumors, making it a potential target for cancer diagnosis, prognosis, and treatment.

#### Medulloblastoma

The most frequent kind of malignant brain tumor in children is medulloblastoma, It originates from the cerebellum, which is located at the lower back of the brain [66]. FOXR2 was implicated in the development of CNS-ET such as medulloblastoma [67,68]. A case report study revealed that a neuroblastoma tumor with FOXR2 activation had both neuronal and glial differentiation [45]. Koso et al. suggest that FOXR2 is overexpressed in medulloblastomas and that it promotes the proliferation of granule neuron precursor cells [13]. FOXR2 was found to be an oncogene in medulloblastoma [41]. However, Koso et al. showed that overexpression of FOXR2 in NIH3T3 cells increased granule neuron precursor (GNP) proliferation; Tgif2 and Alx4 had the same effect. These findings provide genetic and functional proof that FOXR2 is related to the SHH subtype of medulloblastoma [13]. Beckmann et al. reported that FOXR2 interacts with N-MYC, enhances the protein stability of C-MYC, and activates FAK/SRC signaling [44]. Jackson et al. discovered that FOXR2 could be an oncogene that drives medulloblastoma, a type of brain tumor [69]. Their findings also suggest new potential targets for treating this disease [69]. FOXR2 upregulation also transformed NIH3T3 cells and increased the proliferation of granule neuron precursors (GNPs), which are involved in tumorigenesis [13]. Medulloblastoma, the most common malignant brain tumor in children, is linked to FOXR2 overexpression, which promotes granule neuron precursor cell proliferation and is associated with the SHH subtype, suggesting FOXR2 as a potential therapeutic target.

#### CNS/peripheral neuroblastoma

Neuroblastoma, a deadly childhood cancer, arises from neural crest cells that contribute to the peripheral nervous system and are linked to early embryonic development of the sympathoadrenal lineage [70]. Łastowska et al. and Korshunov et al. found that FOXR2 was highly expressed in neuroblastoma of the CNS [71,72]. FOXR2 expression can cause and maintain transformation in different types of cells, including CNS and peripheral neuroblastoma cells, as shown by *in vitro* and *in vivo* models [73,74]. CNS-NB-FOXR2 represents a unique subtype of CNS-neuroblastoma characterized by FOXR2 activation and distinct imaging features [19]. The function and mechanism of FOXR2 in both CNS and peripheral neuroblastoma need further investigation. CNS-NB-FOXR2 and peripheral neuroblastoma, despite their similar nomenclature, represent different tumor entities; the former is an embryonal brain tumor, while the latter originates from the adrenal gland. It is critical to distinguish between their origins and not to merge their distinct lineages. Although it is possible that FOXR2 may more readily transform primitive neuroblastic or embryonal cells, any such assertions should clearly differentiate between the two lineages.

#### Pineal parenchymal tumor

Neoplasms in the pineal area can arise from pineal parenchymal cells, residual stem cells, or nearby glia [75,76]. These cells give rise to pineal parenchymal tumors, which make up about 27% of tumors in this region [75]. A meta-analysis of 221 patients with specific types of pineal parenchymal tumors (PPTs) was conducted [77]. These tumors were classified into four groups based on molecular features: PB-miRNA1, PB-miRNA2, PB-MYC/FOXR2, and PB-RB1. The PB-MYC/FOXR2 group showed MYC amplification and FOXR2 overexpression [77]. Pineal area neoplasms, which account for 27% of regional tumors, can originate from various cell types and are classified into four molecular groups, with the PB-MYC/FOXR2 group characterized by MYC amplification and FOXR2 overexpression.

#### Pediatric high-grade gliomas and diffuse midline gliomas

High-grade gliomas (HGG) and diffuse midline gliomas (DMG) cause many cancer-related deaths in children [78]. DMGs are a subtype of HGGs that arise in midline structures of the brain and are associated with a poor prognosis [79]. Tsai et al. investigated the role of FOXR2 in glioma growth in different brain regions, based on genomic analysis that showed FOXR2 expression in both HGGs and midline/brainstem DMG tumors [14]. They also measured FOXR2 expression and associated genetic traits such as single nucleotide and structural variations in a new and existing cohort of pediatric high-grade gliomas and diffuse midline gliomas [14]. They found that FOXR2 was highly expressed in various pediatric cancers, including neuroblastoma, DMGs, and sarcomas [14]. FOXR2 activation may be a cause of tumor formation, as shown by the presence of L1/FOXR2 fusion transcripts, FOXR2 overexpression, and a tumor methylation profile that matched the original pediatric high-grade glioma that occurred two years before [80]. It was also reported that abnormal FOXR2 activation could lead to the methylome profile and cancer in pediatric brain tumors [81]. This activation was due to the gain of the oncogenic FOXR2 promoter in a pediatric brain tumor [81]. In summary, FOXR2 overexpression, linked to genetic variations and abnormal activation, plays a significant role in the development of pediatric high-grade gliomas and diffuse midline gliomas, contributing to the poor prognosis of these brain tumors.

### Endocrine system

#### Thyroid cancer

Thyroid cancer occurs when malignant (cancer) cells grow in the thyroid gland tissues [82]. FOXR2 expression was high in TC tissues and cell lines. Furthermore, FOXR2 overexpression is associated with tumor aggressiveness and poorer patient outcomes in thyroid cancer [83]. Reducing FOXR2 blocked TC cell migration and invasion by hypoxia-induced reactive oxygen species (ROS) [84]. This effect was partly mediated by the hedgehog pathway [84]. These findings suggested that lowering FOXR2 could inhibit TC cell migration and invasion triggered by hypoxia-driven ROS through the hedgehog pathway [84]. Therefore, FOXR2 might be a potential target for TC treatment.

### Lymphoma and bones and joints

#### Hodgkin lymphoma

Hodgkin lymphoma (HL) is a lymphatic malignancy that affects the immune system. In 2021, an estimated 8830 new cases and 960 deaths from HL were expected in the United States [85]. While FOXR2’s role has been extensively studied in various cancers, its specific involvement in Hodgkin lymphoma (HL) is less clear. However, the FOX family of genes, to which FOXR2 belongs, has been implicated in the pathology of Hodgkin lymphoma. Deregulated FOX genes, including FOXR2, have been noted in the context of HL, suggesting that FOXR2 might contribute to the disease’s molecular pathology [83]. FOXR2 expression was also elevated in HL tissues and promoted tumor growth in HL cell lines [86].

#### Osteosarcoma

Osteosarcomas are bone cancers that arise from immature bone-forming cells [87]. Osteosarcoma tissues and cell lines express high levels of FOXR2 [14]. In osteosarcoma, miR-202 acts as a tumor suppressor by targeting FOXR2. This microRNA suppresses cell proliferation by downregulating FOXR2 expression, indicating that FOXR2’s oncogenic activity can be mitigated by miR-202 [40]. FOXR2 plays a significant oncogenic role in osteosarcoma by promoting cell proliferation. Its regulation by miR-202 and potential as a therapeutic target highlights the importance of understanding FOXR2’s mechanisms in cancer biology. Further research could lead to the development of targeted therapies aimed at inhibiting FOXR2 to treat osteosarcoma effectively.

## Regulation of FOXR2 through Different Pathways

FOXR2 overexpression in ovarian cancer enhances angiogenesis and triggers the hedgehog signaling pathway, which partly explains the aggressiveness of cancer cells [36]. The hedgehog signaling pathway may be essential for FOXR2-induced tumor growth [36]. FOXR2 was identified as a new target of miR-1252, which was sequestered by circCELSR1. This reduced the expression of FOXR2 [37]. FOXR2 promotes tumor growth by activating MYC transcription in different human cancer cell lines and tissues, such as breast, lung, and liver. FOXR2 downregulation suppresses non-small cell lung cancer cell growth and invasion through the Wnt/β-catenin signaling pathway [32]. FOXR2 also regulates MYC/MYCN stability and activates various pathways in different contexts, such as the FAK/SRC signaling, SHH activation, p27 pathway, and epithelial-to-mesenchymal transition [11,13,18,44]. Moreover, by affecting the hedgehog pathway, the down-regulation of FOXR2 blocks thyroid cancer cell invasion and migration caused by hypoxia-induced ROS.

## FOXR2 Prognostic and Therapeutic Potential

The potential of FOXR2 as a prognostic biomarker is indicated by its association with patient outcomes (Table 1), enabling clinicians to identify patients with an increased risk of disease progression or poor survival. Detecting FOXR2 expression or specific alterations in cancer tissues may also serve as a diagnostic tool for particular cancer types or subtypes, allowing for more precise and tailored treatment strategies [35]. FOXR2 is abnormally expressed in a number of malignancies and is highly associated with carcinogenesis [18]. However, cancer patients with higher FOXR2 expression levels often have a better overall survival rate (OS). This is the case for glioma [28], primary neuroblastomas [18], non-small cell lung cancer [32,50], endometrial adenocarcinoma [40], pediatric brain tumors [71], thyroid cancer [86], breast cancer [35], and epithelial ovarian cancer [88]. FOXR2 expression identifies pediatric cancer patients with low 10-year overall survival rates of 53% to 59%, regardless of other risk factors [18]. FOXR2 is essential for these cancers, as shown by FOXR2 knockdown in neuroblastoma cell lines that reduced cell cycle, growth, survival, and MYCN protein levels [18]. Many breast, lung, and liver cancer cell lines and tumor samples express high levels of FOXR2, and lowering FOXR2 expression in a xenograft model slows down tumor growth [11].

**Figure 6:**
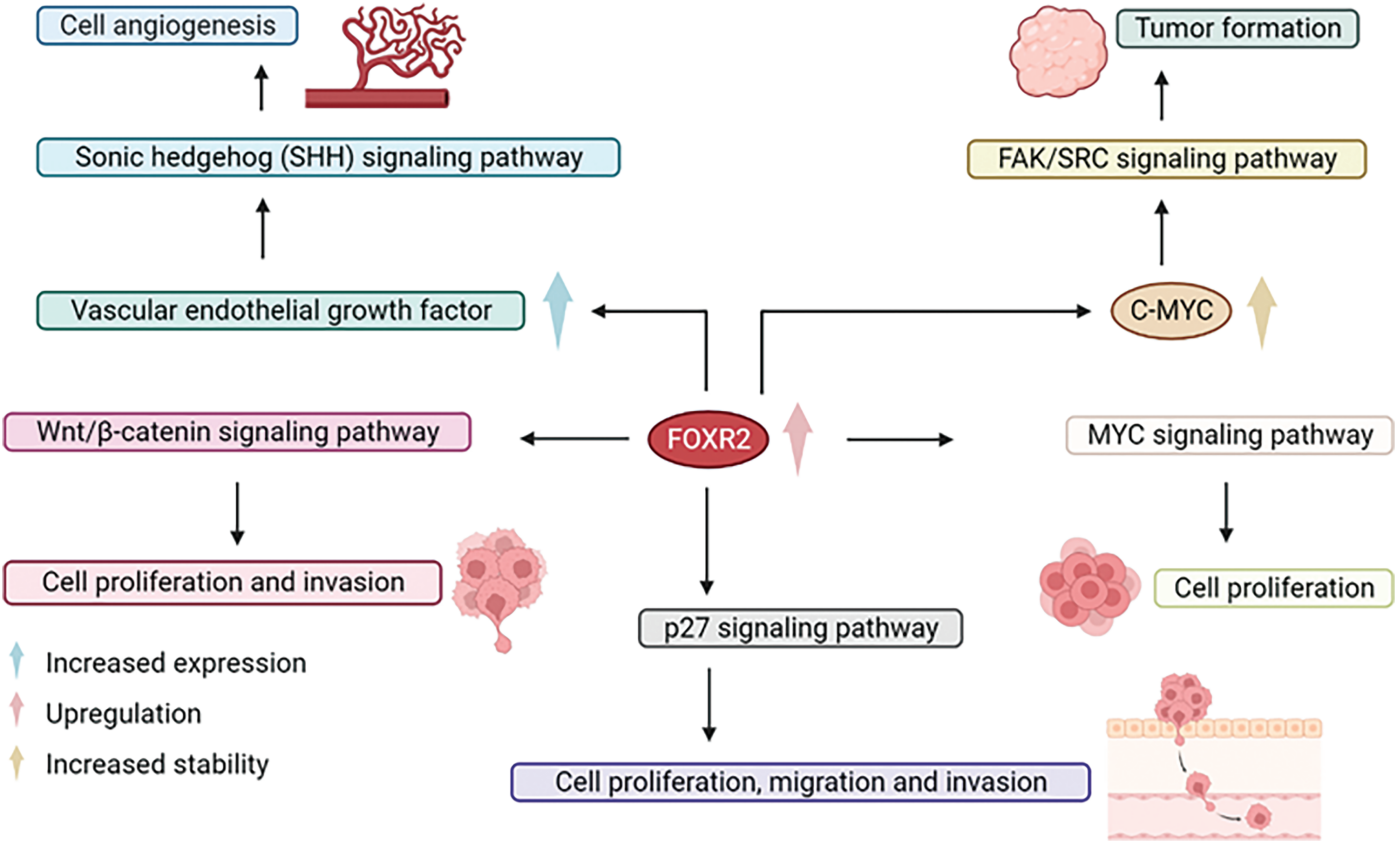
Potentials of FOXR2 in strategic cancer therapy. Targeting FOXR2 activity presents a promising approach for treating cancers where it’s a key player.

In high-grade tumors, there was a strong relationship between the FOXR2 gene expression profile and EMT-related markers [88]. High FOXR2 levels are linked to larger tumors and lymph node metastases in breast cancer patients [35]. FOXR2 may also possibly be a significant molecular marker in the diagnosis and prognosis of breast cancer [35]. The high expression of FOXR2 is also related to the paclitaxel (PTX)-resistance of ovarian cancer cell lines [37]. Real-time PCR studies showed that PTX-resistant ovarian cancer tissues had more FOXR2 than PTX-sensitive ones. FOXR2 may affect the outcome of patients with PTX-resistant ovarian cancer [37]. The TF FOXR2 promotes prostate cancer cell migration, proliferation, and invasion by increasing MMP-2 expression and activity, decreasing p27 expression and nuclear localization, and activating the β-catenin/cyclinD1/c-Myc pathway, as shown by FOXR2 knockdown experiments [39]. Moreover, the involvement of FOXR2 in critical cancer-related processes, such as cell proliferation, apoptosis, and DNA damage response, highlights its potential as a therapeutic target [31,47]. Targeting FOXR2 activity presents a promising approach for treating cancers where it’s a key player. Developing therapeutic agents like small molecules or antibodies that focus on FOXR2, or its related pathways could be effective. Specifically, in prostate cancer, FOXR2 is vital for cell growth and spread, functioning through the suppression of the Wnt/β-catenin signaling pathway, which is instrumental in the disease’s advancemen (Fig. 6) [39]. Therefore, FOXR2 could be a potential therapeutic target for treating prostate cancer [39].

In short, FOXR2 is a significant prognostic biomarker and therapeutic target in various cancers. Its expression is linked to patient outcomes, aiding in the identification of those at higher risk of disease progression or poor survival. FOXR2 is abnormally expressed in several malignancies, including glioma, neuroblastomas, non-small cell lung cancer, and breast cancer, often correlating with better overall survival. It plays a crucial role in cancer-related processes like cell proliferation and apoptosis, making it a promising target for new therapies. High FOXR2 levels are associated with larger tumors, metastases, and drug resistance, particularly in breast and ovarian cancers, highlighting its potential for more precise diagnosis and treatment strategies.

## Potential of the FOXR2 in Personalized Medicine

Personalized medicine is an emerging approach in cancer treatment that takes into account the unique genetic and molecular characteristics of individual tumors [89]. The identification of FOXR2 as a key player in specific cancer types or subgroups contributes to the development of such personalized medicine strategies [14].

By understanding the distinct genetic makeup and molecular features of a patient’s tumor, clinicians can design targeted treatment plans that exploit specific vulnerabilities, such as aberrant FOXR2 expression [14]. This tailored approach allows for more effective and precise interventions, leading to improved treatment outcomes and potentially reducing side effects.

Molecular therapy models for targeting FOXR2 in cancer treatment include RNA interference (RNAi) therapy, CRISPR-Cas9 gene editing, small molecule inhibitors, and antibody-based therapies. RNAi uses siRNAs or shRNAs to silence FOXR2, reducing its expression and potentially inhibiting tumor growth [90]. CRISPR-Cas9 allows precise gene editing to knock out FOXR2, offering insights into its role in cancer and serving as a potential therapeutic strategy [91]. Small molecule inhibitors can specifically block FOXR2 activity, while monoclonal antibodies can target FOXR2 or its signaling pathways [92]. Each method faces challenges such as delivery efficiency and off-target effects, but advancements in delivery systems, gene editing technologies, and drug design are expected to overcome these obstacles. Continued research and innovative strategies are crucial for translating these models into clinical practice, aiming to improve outcomes for patients with FOXR2-driven cancers. FOXR2 is a significant target in cancer pharmacology due to its role as an oncogene in various cancers, including gliomas and prostate cancer. Drug discovery efforts focus on identifying small molecule inhibitors that specifically bind to and inhibit FOXR2 activity [92]. Preclinical studies involve *in vitro* and *in vivo* testing to assess these compounds’ efficacy, pharmacokinetics, and mechanisms of action. Clinical development progresses through Phase I to III trials to evaluate safety, efficacy, and optimal dosing in patients [14]. Challenges include validating FOXR2 as a universal target, addressing potential drug resistance, and personalizing treatment based on FOXR2 expression. Successful strategies can lead to new, targeted cancer therapies that improve patient outcomes.

Moreover, the use of FOXR2 as a prognostic or predictive biomarker can help stratify patients based on their risk of disease progression or response to specific treatments [35]. This information can guide clinical decision-making and enable the selection of the most appropriate treatment regimen for each patient, optimizing the potential for successful outcomes.

In conclusion, the integration of FOXR2 and other key molecular markers into personalized medicine strategies has the potential to transform cancer care by providing tailored treatment options that address the unique characteristics of each patient’s disease. This approach not only improves treatment outcomes but also paves the way for the development of novel therapeutic strategies targeting crucial molecular players like FOXR2.

## Conclusions and Perspectives

FOX genes, including FOXR2, encode TFs essential for various biological processes such as development and immune regulation. FOXR2 is increasingly recognized as an oncogene associated with cancer progression, metastasis, and drug resistance. Despite its prevalence in cancer lineages, the epigenetic triggers of FOXR2 activation remain poorly understood, necessitating tumor-specific insights before clinical translation. Unraveling FOXR2’s epigenetic activation and expanding its transcriptional targets could pave the way for novel therapeutic strategies in both adult and pediatric malignancies. While FOXR2 holds promise as a therapeutic target and biomarker, challenges persist in elucidating its regulatory mechanisms, validating it as a biomarker, and developing targeted therapies. High-throughput techniques like single-cell sequencing and epigenetic analyses are poised to illuminate FOXR2’s molecular interactions and structural characteristics, crucial for drug development. FOXR2 shows promise as a specific and sensitive biomarker for certain cancers, but challenges remain in heterogeneous expression, detection method standardization, and clinical validation. Combining FOXR2 with other biomarkers and advanced molecular techniques can enhance diagnostic accuracy, making it a valuable tool in cancer diagnosis and management.

While FOXR2 is known to be an epigenetically regulated oncogene that activates ETS transcriptional circuits, the precise mechanisms by which it induces tumor formation are not fully understood. This lack of detailed mechanistic insight poses a challenge for developing targeted therapies [14]. Much of the current understanding of FOXR2’s role in cancer comes from preclinical studies. There is a need for more comprehensive clinical data to validate FOXR2 as a therapeutic target and to understand its role across different cancer types. The retrospective nature of some studies and variability in treatment protocols also limit the ability to draw definitive conclusions about its clinical implications [18,93]. FOXR2’s role and expression levels vary across different cancer types, which complicates the development of a one-size-fits-all therapeutic approach. Understanding these variations is essential for designing effective, tailored treatments.

Last but not least, integrating beneficial microbes into the therapeutic landscape for FOXR2-driven cancers presents a promising future direction [94,95]. By leveraging the modulatory effects of the microbiome on the immune system and cancer progression, new therapeutic strategies can be developed. Ongoing research into the interactions between FOXR2 and beneficial microbes, along with advancements in microbial engineering, holds the potential to enhance cancer treatment and improve patient outcomes [96–98].

This review article has limitations such as data heterogeneity, lack of clinical validation, and variability in FOXR2 expression across different cancer types. The precise epigenetic mechanisms of FOXR2 activation remain unclear, complicating the development of targeted therapies. Standardizing detection methods and overcoming the retrospective nature of some studies are additional challenges. Technological advancements like single-cell sequencing and epigenetic analyses are promising but still evolving. Addressing these limitations is essential for fully leveraging FOXR2’s potential as a therapeutic target and biomarker in cancer treatment.

In conclusion, FOXR2 is a promising target in cancer pharmacology, but several research limitations need to be addressed to fully capitalize on its therapeutic potential. Understanding its mechanisms, developing specific inhibitors, and improving clinical data are critical steps toward making FOXR2-targeted therapies a reality. Ongoing research and technological advancements hold promise for overcoming these challenges and improving outcomes for patients with FOXR2-driven cancers.

## Data Availability

None.
